# High-bandwidth multimode self-sensing in bimodal atomic force microscopy

**DOI:** 10.3762/bjnano.7.26

**Published:** 2016-02-24

**Authors:** Michael G Ruppert, S O Reza Moheimani

**Affiliations:** 1 School of Electrical Engineering and Computer Science, The University of Newcastle, Callaghan, NSW, 2308, Australia; 2 Department of Mechanical Engineering, The University of Texas at Dallas, Richardson, TX, 75080, U.S.A.

**Keywords:** atomic force microscopy, charge sensing, feedthrough cancellation, multimode sensor, piezoelectric cantilever, self-sensing

## Abstract

Using standard microelectromechanical system (MEMS) processes to coat a microcantilever with a piezoelectric layer results in a versatile transducer with inherent self-sensing capabilities. For applications in multifrequency atomic force microscopy (MF-AFM), we illustrate that a single piezoelectric layer can be simultaneously used for multimode excitation and detection of the cantilever deflection. This is achieved by a charge sensor with a bandwidth of 10 MHz and dual feedthrough cancellation to recover the resonant modes that are heavily buried in feedthrough originating from the piezoelectric capacitance. The setup enables the omission of the commonly used piezoelectric stack actuator and optical beam deflection sensor, alleviating limitations due to distorted frequency responses and instrumentation cost, respectively. The proposed method benefits from a more than two orders of magnitude increase in deflection to strain sensitivity on the fifth eigenmode leading to a remarkable signal-to-noise ratio. Experimental results using bimodal AFM imaging on a two component polymer sample validate that the self-sensing scheme can therefore be used to provide both the feedback signal, for topography imaging on the fundamental mode, and phase imaging on the higher eigenmode.

## Introduction

Emerging methods in multifrequency atomic force microscopy (MF-AFM) rely on the detection and excitation of higher order eigenmodes of a microcantilever [1–3] and as such, present a number of practical challenges to cantilever instrumentation. Both high-bandwidth cantilever actuation and deflection sensing are necessary, ideally without distorting the frequency response of the cantilever and involving a minimum amount of external equipment. For example, the commonly used piezoelectric actuator at the base of the cantilever leads to a highly distorted frequency response with numerous resonances which renders the identification and subsequent analysis of higher eigenmodes exceedingly difficult.

To circumvent this problem, integrated actuation such as magnetic [4], photothermal [5], resistive thermal [6], ultrasonic [7] or through a piezoelectric layer [8] have been employed. Among the sensing techniques to detect the cantilever oscillations, the optical beam deflection (OBD) method [9] remains the most widely used approach mostly due to its low noise characteristics. However, its limitations such as frequent laser alignment, imaging artifacts due to optical interferences [10] and limited bandwidth requiring custom-built read-out electronics [11–12] have led to the development of numerous integrated sensing approaches. These include capacitive [13], piezoresistive [14], piezoelectric [15] and magnetoresistive [16] sensing.

A common drawback of self-sensing approaches applied to microelectromechanical systems (MEMS) is the fact that drive and sense electrodes share a common node (the MEMS electrical network) resulting in a potentially large feedthrough path from actuation to sensing [17]. If not properly accounted for, this feedthrough can almost entirely conceal the signal originating from the motion of the structure and is especially dominant if the same transduction principle is used for both actuation and sensing. Recently, the authors proposed two reciprocal self-sensing schemes for tapping-mode atomic force microscopy (TM-AFM) utilizing charge sensing and charge actuation respectively [18–19], using a single piezoelectric layer. The proposed techniques enable the elimination of the piezoelectric base actuator and the OBD sensor from the cantilever instrumentation setup, avoiding tedious laser alignment and distorted frequency responses. In this contribution, we demonstrate that the self-sensing method can be extended to MF-AFM techniques such as bimodal imaging by measuring the charge simultaneously at multiple higher eigenmodes. However, the individual resonances are heavily buried in feedthrough originating from the piezoelectric capacitance which yields a dynamic range of less than 1 dB at the resonant modes. In order to recover these modes for subsequent application in MF-AFM, two parallel analog feedforward compensators are employed to cancel the feedthrough at each eigenmode leading to a substantial increase in dynamic range. We demonstrate that on the higher eigenmode, a two order of magnitude increase of sensitivity is achieved due to the large deflection to strain sensitivity. The applicability of the multimodal self-sensing principle is verified by bimodal AFM experiments to obtain qualitative phase contrast on the higher eigenmode when imaging a soft polymer blend.

## Modeling

### Piezoelectric constitutive laws

**Figure 1:**
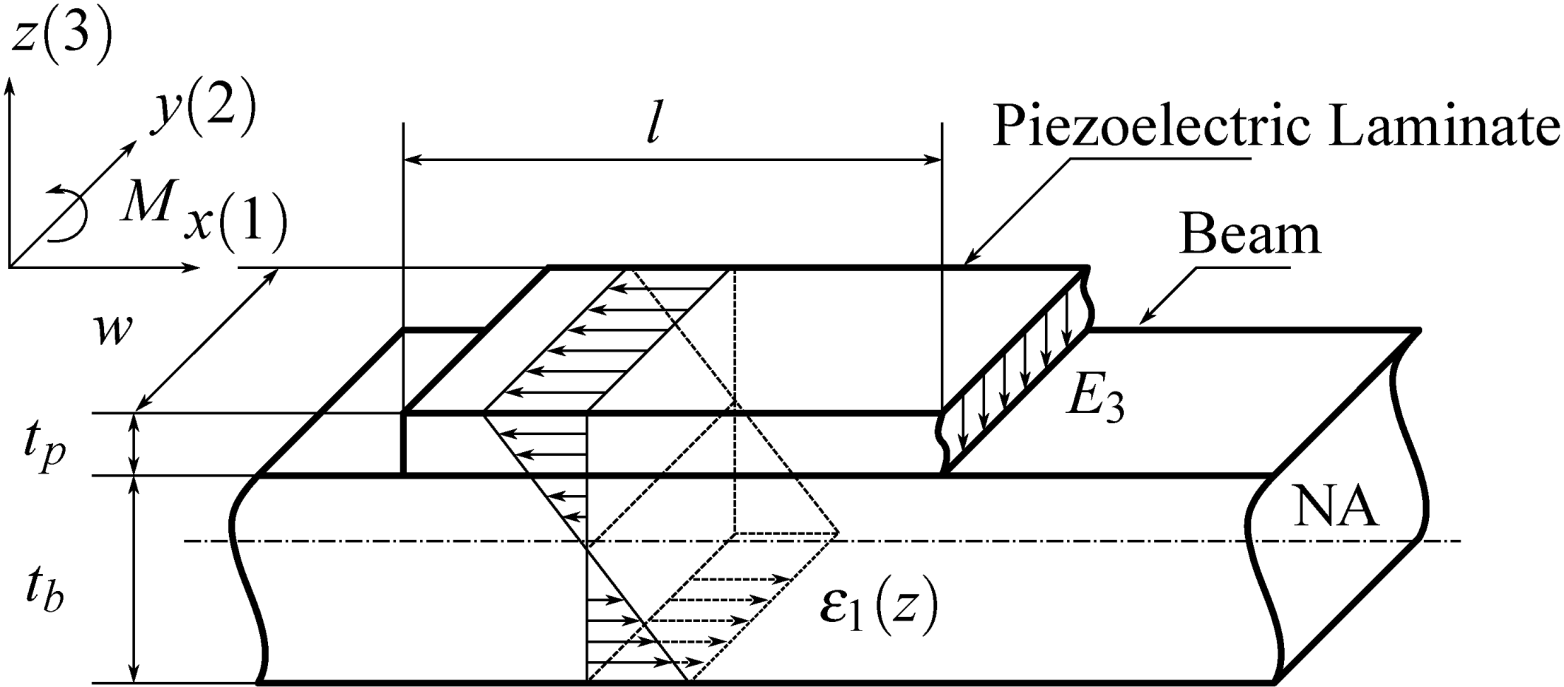
Simplified cross-section schematic of a beam with bonded piezoelectric layer. An electric field *E*_3_ applied to the piezoelectric layer causes a cantilever deflection in the *z*-direction. Conversely, a deflection in the *z*-direction causes stress in the *x*-direction ε_1_(*x*), which leads to a charge accumulation on the piezoelectric layer.

By sputtering a piezoelectric layer to the surface of a cantilever, a transducer with inherent self-sensing capabilities is obtained. The electromechanical equations describing the independent variables applied stress σ [N/m^2^] and applied electrical field *E* [V/m] and the dependent variables resulting strain ε [m/m] and resulting electrical displacement *D* [C/m^2^] within a piezoelectric material are governed by the IEEE standard on piezoelectricity [20]. They are usually written in compact matrix notation such that redundant and symmetrical terms are accounted for. By convention of the axis defined in Figure 1, an electric field or a deflection in the (3)-direction causes normal stress in the (1)-direction [21]. Then, the constitutive equations reduce to two scalar equations

[1]
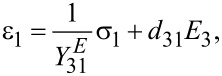


[2]
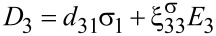


with Young’s modulus *Y* [N/m^2^], piezoelectric *d* [m/V] and dielectric ξ [F/m] material constants. The superscripts *E* and σ indicate that these constants are measured during constant electrical field (electrodes short-circuited) and constant stress (electrodes open-circuited), respectively. Here, Equation 1 states that the total strain is the sum of the mechanical strain due to mechanical stress (passive) and the strain caused by applying an electrical field (active) and therefore describes the transducer if used as an actuator, i.e., the converse piezoelectric effect. On the other hand, Equation 2 states that the total electrical displacement is the sum of induced electrical displacement due to mechanical stress (sensing) and applied electrical field (feedthrough) and therefore describes the transducer if used as a sensor, i.e., the direct piezoelectric effect.

In the following we assume an Euler–Bernoulli beam with homogeneous isotropic linear elastic material with constant cross section and perfect bonding of the piezoelectric layer which is thin and lightweight compared to the beam. The assumption implies a linearly varying strain distribution throughout the beam and enables analytical actuator and sensor equations to be derived [22].

#### Piezoelectric actuator

For a piezoelectric layer with thickness *t_p_*, applying a voltage *V* across the electrodes along the polarization direction, generates the electrical field

[3]
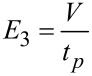


and results in the free strain

[4]
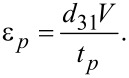


Further, the asymmetrical strain distribution along the (1)-axis in the actuator as shown in Figure 1 can be stated as [23]

[5]
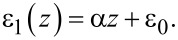


By equating Equation 1 and Equation 5 and using Hooke’s law, the stress distribution in the actuator is found to be

[6]
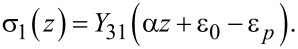


By applying the moment equilibrium around the center of the beam and the force equilibrium along the (1)-axis of the beam, α and ε_0_ can be determined. By further integrating Equation 6 across the beam, the distributed moment as a function of the applied voltage is found to be [23–24]

[7]
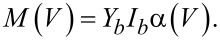


where *I_b_* and *Y_b_* are the moment of inertia and Young’s modulus of the beam and α(*V*) contains geometrical constants of the beam and the piezoelectric layer and is linear in the applied voltage. Thus, a voltage applied to the electrodes results in a bending moment causing the cantilever to deflect.

#### Piezoelectric sensor

With the foregoing assumptions, the stress in the (1)-direction is given by Hooke’s law

[8]
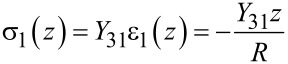


where *R* is the bending radius which can be related to the second derivative along the *x*-axis of the displacement *z*^′′^(*x*,*t*) = 1/*R* to yield

[9]
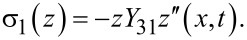


Here, stress is defined to be positive under elongation (tensile stress) and negative under compression (compressive stress). Assuming zero applied electrical field *E*, the electrical displacement *D* due to bending stress is given by Equation 2. Hence, the charge collected on the electrodes located at *z* = *t_b_*/2 + *t_p_* can be determined by integrating the electrical displacement over the electrode area

[10]
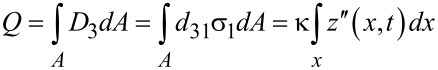


where κ = −*d*_31_(*t_b_*/2 + *t_p_*)*wY*_31_.

#### System model

The transverse deflection of a uniform cantilever (*YI* = const) are governed by the Euler–Bernoulli beam equation, which for the free vibration case are described by the partial differential equation (PDE) [25]

[11]
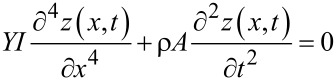


where *Y,I,ρ,A* are Young’s modulus, area moment of inertia, mass density and cross section of the beam respectively. A common approach to solve Equation 11 is the modal analysis approach. Here, it is assumed that the solution can be represented by separable space and time functions representing the mode shape *Z_k_*(*x*) and modal coordinates *q_k_*(*t*)

[12]
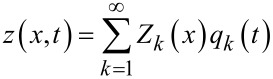


with

[13]
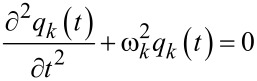


[14]
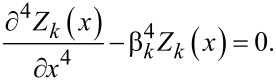


For the case of a homogenous isotropic linear elastic beam with constant cross section, the displacement and strain modeshapes for varying standard boundary conditions can be derived analytically from Equation 14 [25]. Discontinuous beam models have been proposed to take into account varying cross-sections but system identification based on parameter optimization must be employed to reduce modeling errors [26]. In order to arrive at a system-based model and to use frequency domain system identification, a damping term is added to Equation 13 and taking the Laplace transform yields a sum of second order modes to describe the frequency response of the first *n* flexural modes of the beam relating the actuator voltage *V*(*s*) to cantilever deflection *D*(*s*) [24]

**Figure 2:**
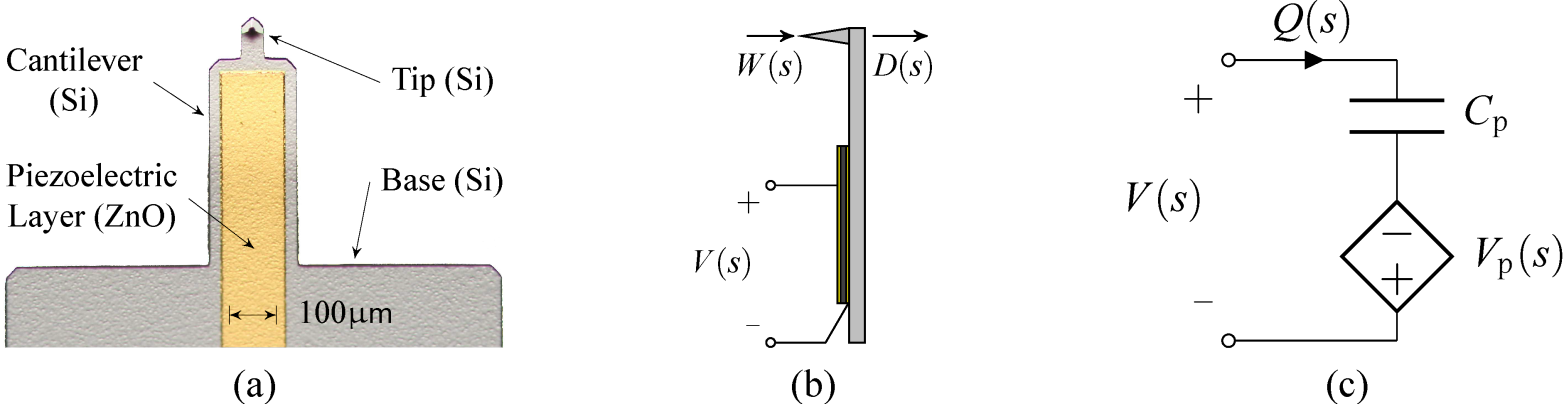
(a) Annotated photo, (b) schematic and (c) electrical circuit model of the piezoelectric cantilever.

[15]
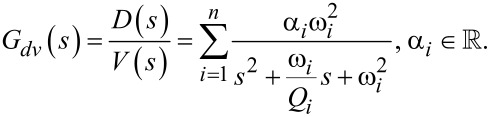


where each second order mode is associated with a specific vibrational mode shape and is characterized in terms of the quality factor *Q_i_*, natural frequency ω*_i_* and gain α*_i_*. Similarly, when a piezoelectric transducer is subjected to mechanical strain it becomes electrically polarized, producing a charge on the surface of the material, described by Equation 10. This direct piezoelectric effect can be modeled as a strain dependent voltage source *V*_p_ in series with a capacitor *C*_p_ as shown in Figure 2c.

While the capacitor sufficiently represents the dielectric properties of the piezoelectric material, this simplified model does not take into account dielectric losses or heat dissipation which can be modeled by adding a resistor in parallel to *V*_p_ and *C*_p_. The model is a simplified version of the Butterworth–van Dyke model as proposed by the IEEE Standard on piezoelectricity [20]. The piezoelectric voltage *V*_p_ can be modeled as the linear combination of the direct excitation voltage *V*(*s*) and a voltage due to the tip–sample force acting as a disturbance *W*(*s*)

[16]
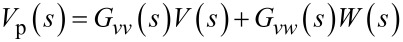


with

[17]
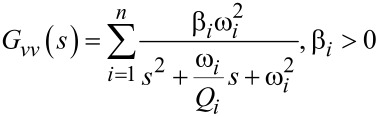


[18]
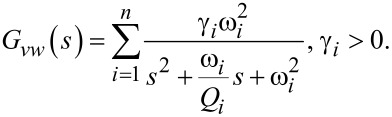


Applying Kirchhoff’s law to Figure 2c, one obtains

[19]
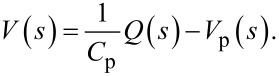


Substituting Equation 16 into Equation 19 yields

**Figure 3:**
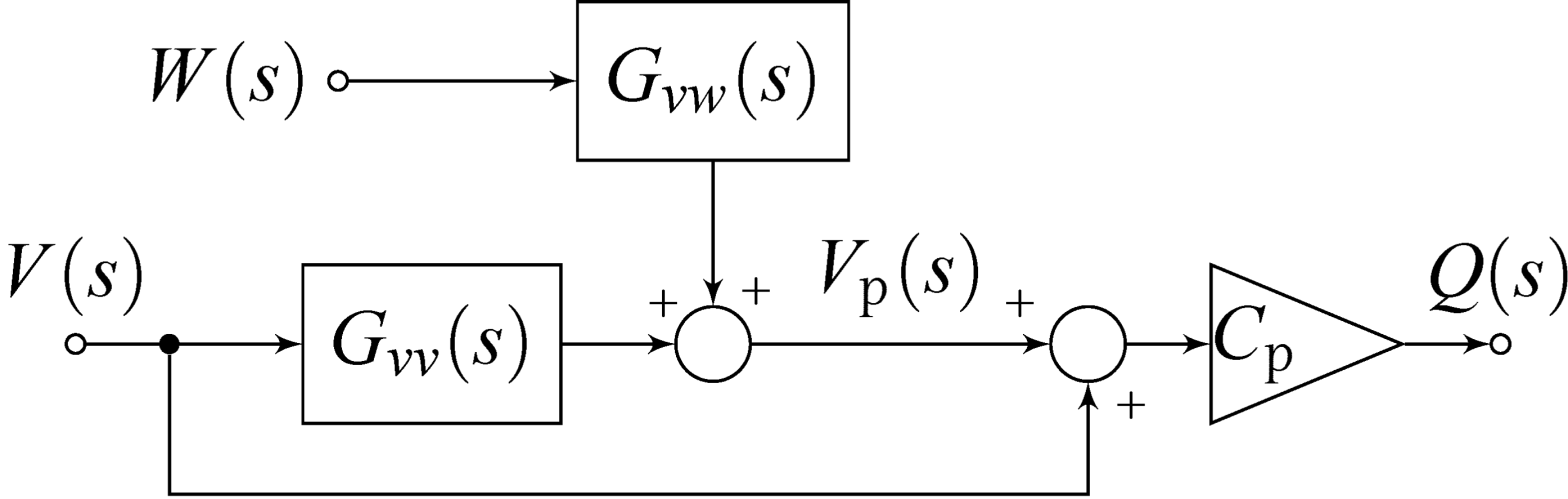
Block diagram representing the transfer function from voltage actuation and tip disturbance to charge in the piezoelectric material.

[20]
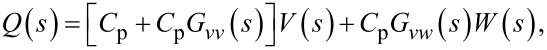


which is illustrated in the block diagram in Figure 3.

We note that the charge in the piezoelectric layer depends on the excitation voltage and the disturbance input but most importantly is dominated by a feedthrough term *C*_p_*V*(*s*). Consequently, the disturbance will remain unnoticed in the charge output if the feedthrough is large. Furthermore, while *G_vw_*(*s*) can be estimated [27–28], it cannot be measured directly. Thus we focus on the system

[21]
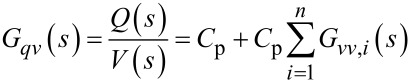


to demonstrate the effect of the feedthrough. Observing that each mode of Equation 15 and Equation 17 only differ by a constant factor, Equation 21 can be rewritten as

[22]
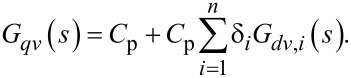


From Equation 22 we conclude that by exciting the cantilever with a voltage and measuring the charge, a deflection estimate of the cantilever can be obtained if the feedthrough term *C*_p_*V*(*s*) can be canceled.

## Results and Discussion

### Implementation

**Figure 4:**
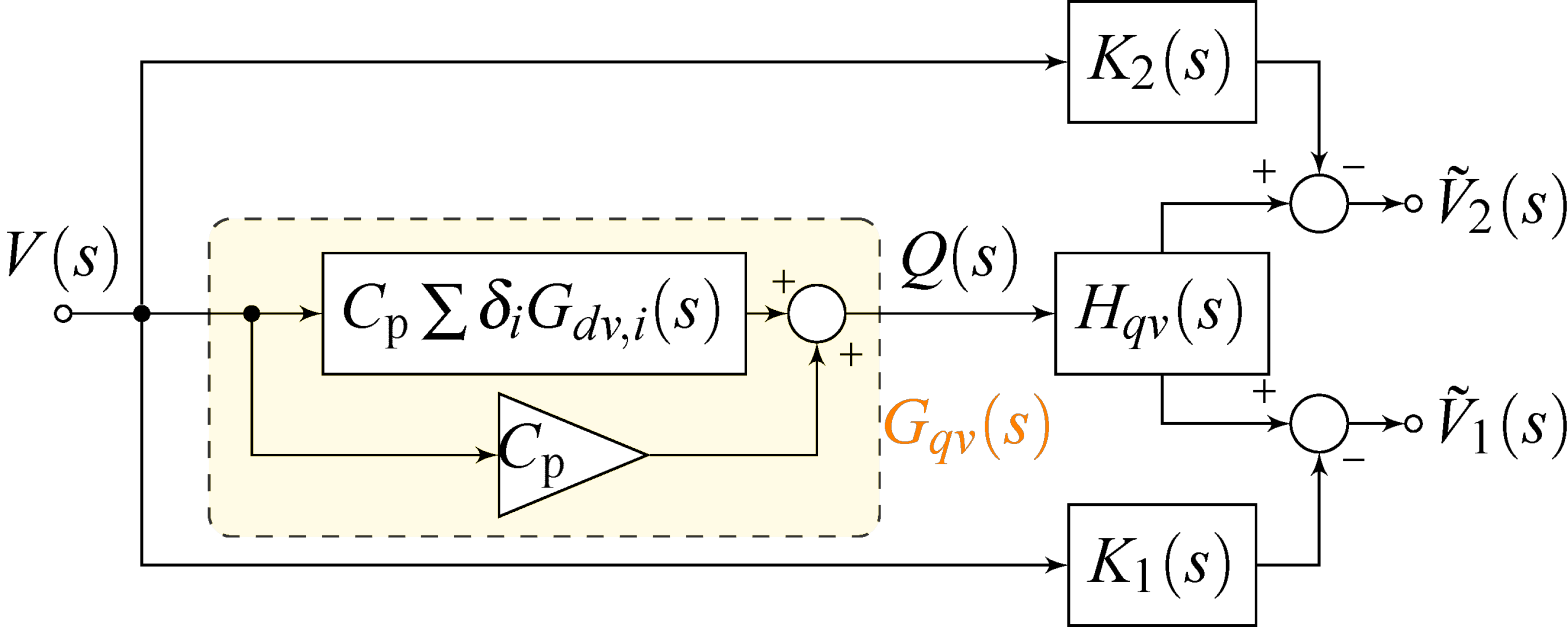
Block diagram of the self-sensing scheme with dual feedforward compensator to cancel the capacitive feedthrough at two resonances.

**Figure 5:**
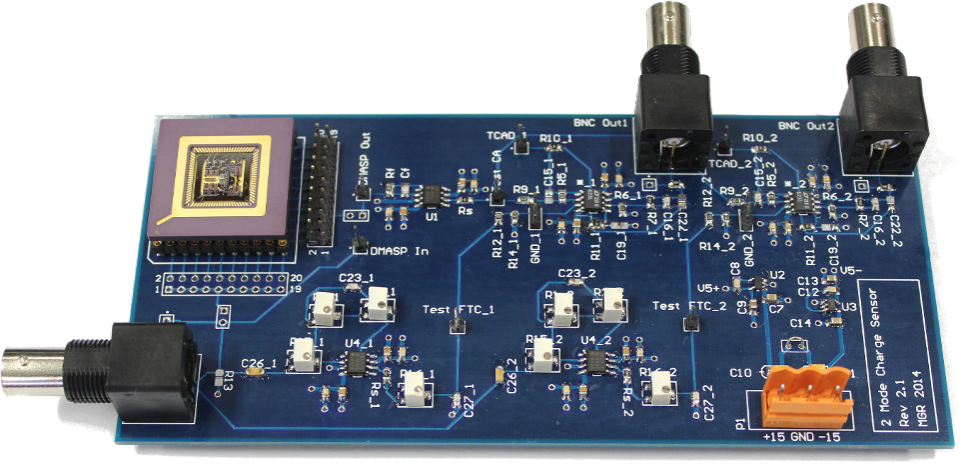
Photo of the implemented PCB circuit for bimodal charge sensing.

The proposed self-sensing scheme was realized using surface-mount high-bandwidth analog components on a printed circuit board (PCB) according to the block diagram shown in Figure 4; a photo of the corresponding implemented circuit is shown in Figure 5. Here, the block *H_qv_*(*s*) models the dynamics of the charge amplifier [18] and the blocks *K*_1_(*s*) = *C*_p_*H_qv_*(*s*) and *K*_2_(*s*) = *C*_p_*H_qv_*(*s*) are feedforward compensators, each containing a model of the charge amplifier stage, to compensate the feedthrough at each resonance. As the charge amplifier can be approximated by a first order high-pass filter in the bandwidth of interest [19], the feedforward compensators will have the same dynamics and can be implemented with simple op-amp circuits. After compensation, the outputs 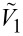 and 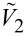 are proportional to the displacement at the respective mode.

### System identification

**Figure 6:**
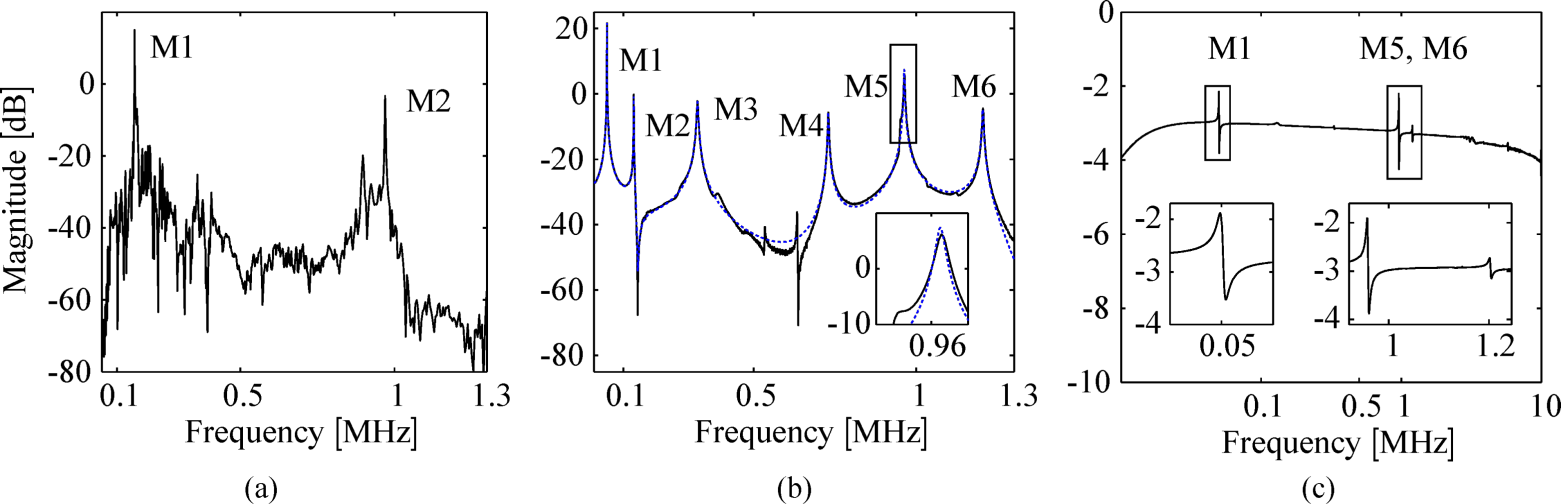
(a) Frequency response measured with the OBD sensor (−) of an NT-MDT NSG01 base-excited cantilever highlighting the first two flexural modes. (b) Frequency response measured with the OBD sensor (−) and identified 12th-order model (−−) of the piezoelectric cantilever highlighting the first six flexural modes. The inset shows a zoomed view of the fifth flexural mode. (c) Frequency response measured with the charge amplifier (−) highlighting the first and fifth flexural modes. The insets show a zoomed view of the first, fifth and sixth modes embedded in capacitive feedthrough.

The AFM cantilever used in this work is a piezoelectric self-actuated silicon microcantilever described in section Modeling. Compared to a standard base excited cantilever whose frequency response is shown in Figure 6a, the piezoelectric cantilever has closely spaced eigenmodes due to the stepped geometry [29] and its frequency response is not distorted by additional actuator dynamics owing to the integrated actuation. The clean nature of the frequency response data, obtained by performing a sinusoidal sweep (Zürich Instruments HF2LI lock-in amplifier), allows for the use of frequency domain subspace identification [30] to obtain a 12-order state space model for the first six eigenmodes of the cantilever. The model along with the measured data is shown in Figure 6b where only the flexural modes have been included in the model (the torsional modes, noticeable between *M*3 and *M*4 as well as in the vicinity of *M*5, have been neglected). From the model, the fixed structure form (Equation 15) for *n* = 6 is calculated with parameters shown in Table 1.

**Table 1:** Parameters of the fixed structure model.

M	shape	*f_i_* [kHz]	*Q_i_*	α*_i_*	*C*_p_ [pF]	δ*_i_*

1	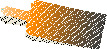	50.1	230	0.054	20.48	0.015
2	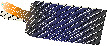	132.4	240	−0.004	—	—
3	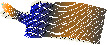	327.9	86	−0.008	—	—
4	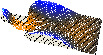	729.3	264	−0.0005	—	—
5	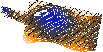	962.5	322	0.004	19.82	0.17
6	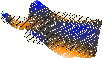	1203.9	335	−0.002	19.71	−0.08

The experimentally obtained voltage to charge frequency response is shown in Figure 6c. We note that the first and fifth modes, while almost entirely buried in feedthrough, show nearly equal gains at the resonance peaks compared to the voltage to deflection frequency response shown in Figure 6b. This is due to the large deflection to strain sensitivity on the higher mode which can be exploited by using a charge sensor.

In order to experimentally verify the model Equation 22, a parameter optimization procedure is employed to fit the model Equation 22 to the experimentally obtained voltage to charge frequency response shown in Figure 6c. The optimization method aims to minimize the difference in magnitude and phase of the measured transfer function and Equation 22. The resulting parameters are also shown in Table 1. We note that the optimization procedure did not converge for the second, third and fourth mode due to the excessive amount of feedthrough. The differences in the estimated feedthrough of each mode is due to numerical rounding occurring when scaling the optimization parameters back to real world quantities (pF) and due to slight variations in the passive components of the analog implementation. Additionally, knowing that the capacitance is an inherent property of the piezoelectric layer, an impedance analyzer such as the Keysight E4990A was used to measure *C*_p_ and the obtained value of 20.27 pF adequately matches the estimation.

### Feedthrough cancellation

**Figure 7:**
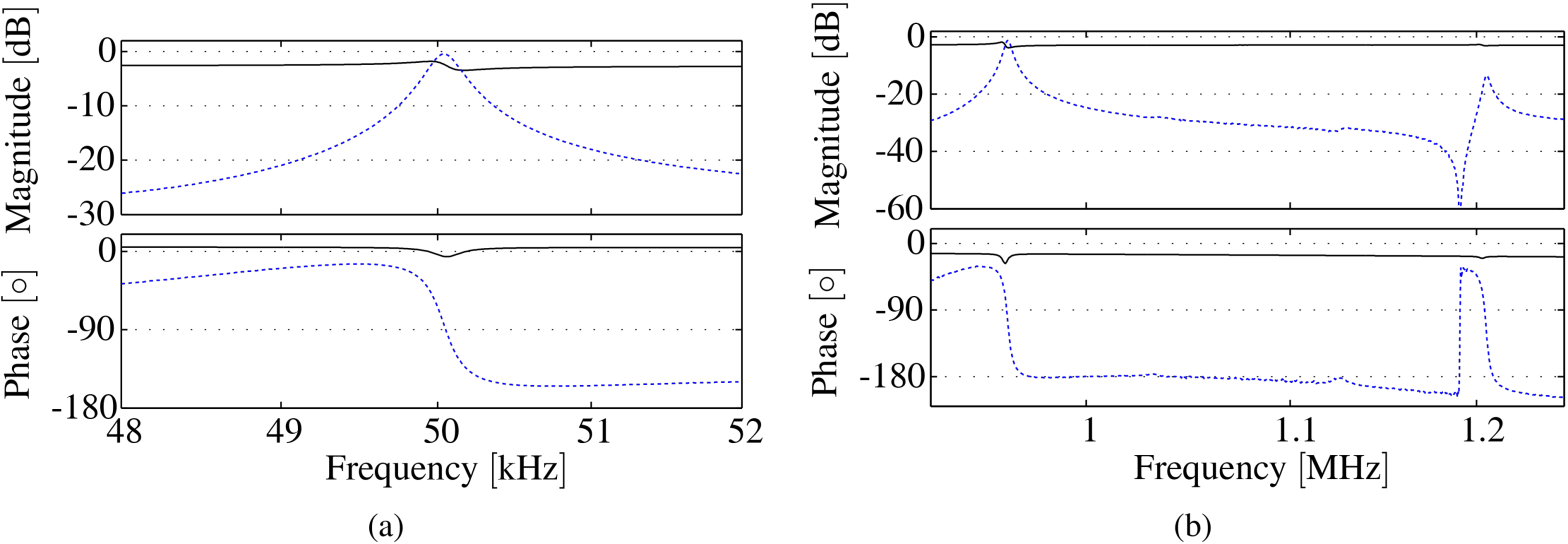
(a) Frequency response of the first flexural mode measured with the charge sensor before (−) and after feedthrough cancellation (−−). The dynamic range has been increased from 0.7 to 25 dB. (b) Frequency response of the fifth and sixth flexural modes measured with the charge sensor before (−) and after feedthrough cancellation (−−). The dynamic range has been increased from 0.9 to 26 dB.

The first and the fifth modes are clearly visible in the frequency response shown in Figure 6c albeit excessively buried in capacitive feedthrough. In order to use the charge sensor for dynamic mode AFM, the eigenmodes need to be recovered from the capacitive feedthrough. Here, an analog feedforward compensation method was employed based on the block diagram shown in Figure 4. It can be seen in Figure 7a how this compensation method leads to an increase in dynamic range around the first resonance from 0.7 dB to 25 dB. Similarly, it can be seen in Figure 7b how the dynamic range around the fifth resonance frequency is increased from 0.9 to 26 dB. Due to slight component mismatches which leads to a phase mismatch, the feedthrough is not compensated entirely which can be seen in the phase response. However, the de-embedded eigenmodes have enough dynamic range to be suitable for bimodal AM-AFM imaging as will be discussed in section Bimodal AFM application.

### Sensor sensitivity

The optical lever method measures the bending angle of the cantilever at the measurement position rather than the displacement [9,31]. As such, the voltage output from the OBD sensor has to be calibrated individually for each eigenmode in order to obtain a deflection measurement from the sensor output [32–33], which is usually done by performing an approach and retract curve on a stiff sample. Assuming that the *z*-axis actuator has been calibrated beforehand, the vibrational inverse optical lever sensitivity (invOLS) can be found by calculating the slope of the linear region of the amplitude versus distance curve [32]. While this approach is common practice for the fundamental mode, it is not feasible for higher eigenmodes, due to their increased dynamic stiffnesses and associated small free-air amplitudes. As such, the sensor sensitivities are calibrated by comparing the sensor outputs for a given drive voltage and comparing it to the displacement measurements obtained from a laser-doppler vibrometer (LDV) (Polytec MSA-100-3D). For the cantilever used in this work and the NT-MDT NTegra AFM system, the inverse optical lever sensitivity for the first mode was found to be

[23]
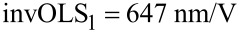


and

[24]
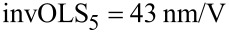


for the fifth mode. Notice, that the sensitivity on the higher eigenmode is an order of magnitude better than on the fundamental mode due to measurement of slope. Similarly, the inverse charge amplifier sensitivity (invCAS) for the first mode was determined to be

[25]
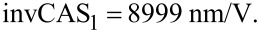


Notice, that this value is significantly higher than the one obtained with the OBD sensor but it can be lowered if subsequent gain stages are employed at the expense of introducing additional sensor noise. However, on the fifth mode we obtain an invCAS of

[26]
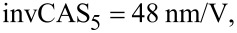


which is more than two orders of magnitude better than on the fundamental mode. This highlights the increased deflection to strain sensitivity on the higher mode which was already noticed from Figure 6c. On the fifth mode, the strain sensor produces the same output for a much smaller deflection, yielding a much larger sensitivity.

### Noise analysis

**Figure 8:**
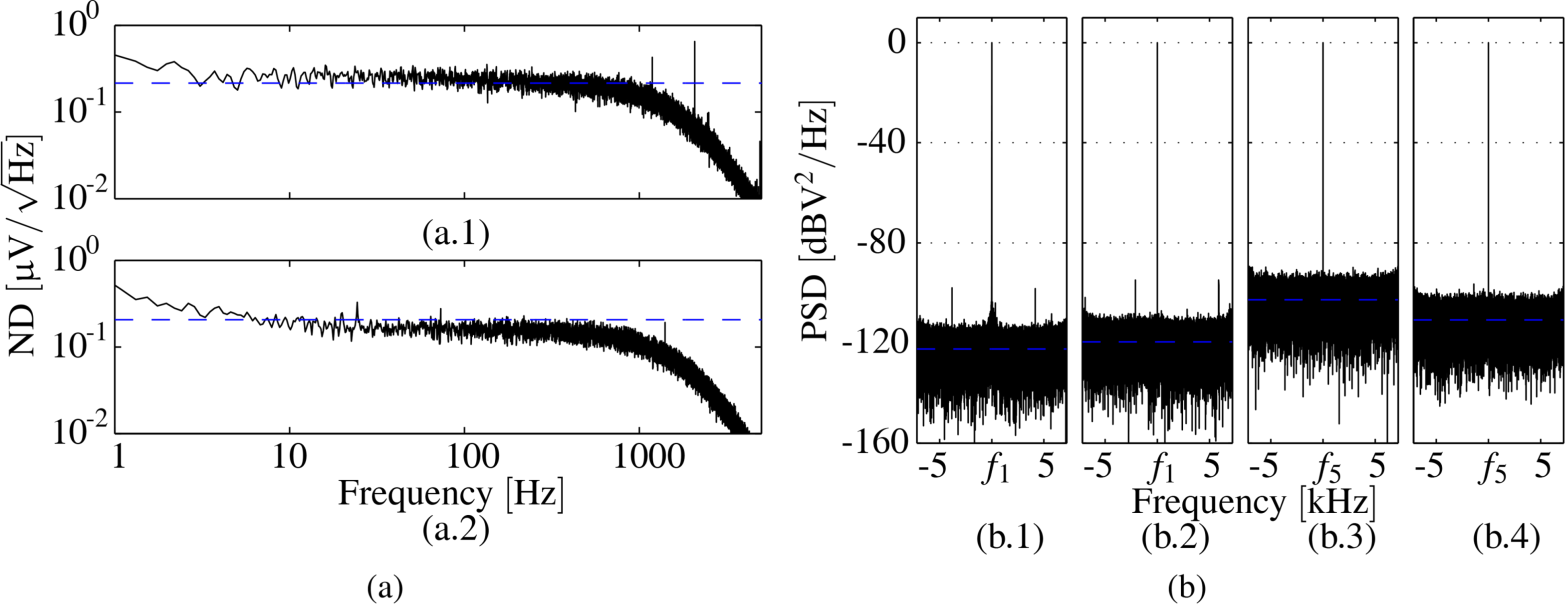
(a) Voltage noise density estimate of demodulated amplitude obtained from LIA with low-pass filter cut-off frequency of *f*_c_ = 1 kHz measured with charge sensor (−) and total integrated noise (−−) of (a.1) first mode and (a.2) fifth mode. (b) Zoom FFT of the deflection estimate on the fundamental mode from (b.1) OBD sensor and (b.2) charge sensor with a span of 14.4 kHz around the resonance. Zoom FFT of the deflection estimate on the fifth mode from (b.3) OBD sensor and (b.4) charge sensor with a span of 14.4 kHz around the resonance.

The noise performance of cantilever deflection sensors used in dynamic AFM is commonly evaluated with the deflection noise density acquired from thermally induced vibrations. However, this method is only suitable for the fundamental mode as higher eigenmode deflections due to Brownian motion decrease rapidly [34]. For the cantilever used in this work, the thermally induced vibration amplitude corresponding to the first mode is below the sensitivity of the charge sensor associated with that mode. As the use of the charge sensor in amplitude modulation AFM always requires demodulation, we state the total integrated noise from the voltage noise density (ND) plot and standard deviation (RMS noise) of the amplitude obtained from a lock-in amplifier (LIA) (HF2LI Zürich Instruments) and compare the measurements with the OBD sensor. The cantilever is actively driven at each mode, resulting in a deflection of 253 nm on the first mode and 1.62 nm on the fifth mode. A 4th-order low-pass filter with cut-off frequency of *f*_c_ = 1 kHz is used in the LIA. The ND estimates are shown in Figure 8a which are obtained from the time-domain demodulated amplitude signals sampled at 28.8 kHz using Welch’s segment averaging estimator with 8 sections windowed with the Hamming window. The results are summarized in Table 2.

**Table 2:** Noise performance of OBD and charge sensor.

	OBD M1	CA M1	OBD M5	CA M5

ND [μV/√Hz]	0.90	0.22	0.70	0.21
ND [fm/√Hz]	585	1940	30.2	9.91
RMS [μV]	30.3	7.20	23.5	6.91
RMS [pm]	19.6	64.8	1.01	0.33

It can be noticed, that the charge sensor shows a lower voltage noise density, but it is inferior to the OBD sensor on the fundamental mode due to the low deflection to strain sensitivity. However, on the fifth mode the large increase in sensitivity results in a deflection noise density of only 9.91 fm/√Hz and RMS noise of 0.33 pm from DC to the equivalent noise bandwidth of the LIA low-pass filter. The values on the fundamental mode are higher than the ones reported for optimized OBD sensor systems using thermal deflection noise density [11]. This is due to the lower sensitivity of the sensor at that mode and the fact that the cantilever is actively driven at resonance. However, the authors believe that the procedure is closer to the actual dynamic AFM application (using lock-in demodulation of actively driven cantilevers) and therefore the values reported are a realistic representation of values obtained during AFM imaging. To qualify the resolution of the overall AFM system, a noise image with the actively driven cantilever in contact with the sample surface should be acquired [35] which takes into account all contributing noise processes.

Additionally, the signal-to-noise-ratio (SNR) is determined from narrowband demodulation (ZoomFFT, HF2LI Zürich Instruments) at a frequency span of 14.4 kHz around the resonance frequency of interest. The results for driving the fundamental mode at an amplitude of 253 nm are presented in Figure 8b.1 and Figure 8b.2, yielding a SNR of the OBD sensor of 122.4 dB and of the charge sensor of 120.0 dB. The results for driving the fifth mode at an amplitude of 1.62 nm are presented in Figure 8b.3 and Figure 8b.4 yielding a SNR of the OBD sensor of 102.8 dB and of the charge sensor of 110.9 dB. The SNR has been calculated from the difference between the fitted noise floor and the peak at the signal of interest. Maximum signal levels have been shifted to 0 dB and the horizontal blue line indicates the fitted noise floor.

### Bimodal AFM application

#### Experimental setup

The experimental setup consists of an unaltered NT-MDT NTegra Prima AFM fitted with a custom cantilever holder to mount the piezoelectric cantilever used in this work. The signal access module (SAM) of the AFM provides the relevant inputs and outputs to change the feedback signal from the OBD sensor measurement to charge measurement. Approach and retract curves as well as all AFM imaging data were recorded using two synchronized Zürich Instrument HF2LI lock-in amplifiers for which custom imaging scripts were written. Therefore, it is possible to obtain AFM images relating to either sensor while *z*-axis feedback is performed on one specific sensor.

The samples under investigation are a TGZ1 silicon calibration grating available from NT-MDT with periodic rectangular features of heights *h* = 21.6 ± 1.5 nm and a blend of polystyrene (PS) and polyolefin elastomer (ethylene-octene copolymer) (LDPE) available from Bruker (PS-LDPE-12M). The PS regions of the sample have elastic modulus numbers around 2 GPa, while the LDPE regions have elastic modulus numbers around 0.1 GPa making it a widely used standard to image material contrast. The scan speed was set to 20 μm/s at an area of 10 μm × 10 μm.

#### Approach curves

**Figure 9:**
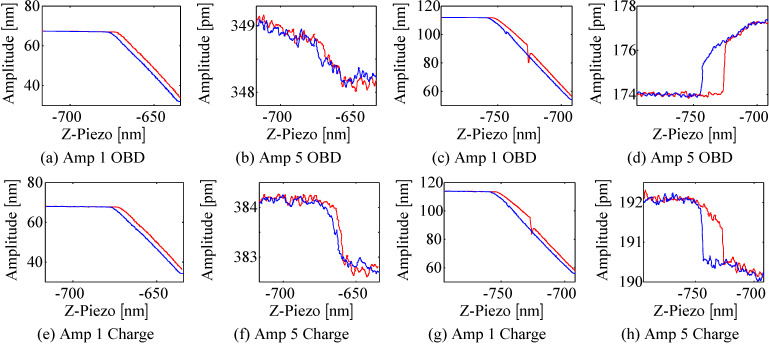
Approach (red, −) and retract (blue, −) curves obtained on a TGZ1 calibration grating with OBD sensor used for feedback: (a)–(d) fundamental and fifth mode amplitude measured with the OBD sensor and (e)–(h) fundamental and fifth mode amplitude measured with the charge sensor.

Approach and retract curves have been performed on the (stiff) TGZ1 calibration grating where the fundamental and the fifth modes are actively driven and the amplitude of the fundamental mode obtained from the OBD sensor is used for *z*-feedback. As can be seen from Figure 9a,b,e,f, the fundamental and higher eigenmode amplitudes measured with either OBD sensor or charge sensor show a similar trend for small drive voltages (free-air amplitudes) which resembles approach and retract curves in one of the two stable branches of the cantilever [36]. However, when the drive voltage of the fundamental mode is increased, the approach curve is characterized by the well known transition between the low and high amplitude branch as can be seen in Figure 9c and Figure 9g. It is worth noting that for this case, the fifth mode amplitudes obtained from the OBD sensor and from the charge sensor form a hysteresis loop and more significantly show inverse behavior for small separations (compare Figure 9d and Figure 9h). As such, the deflection of the fifth mode increases and the strain decreases when the cantilever oscillation state jumps from one amplitude branch into the other.

#### Imaging TGZ1 calibration grating

**Figure 10:**
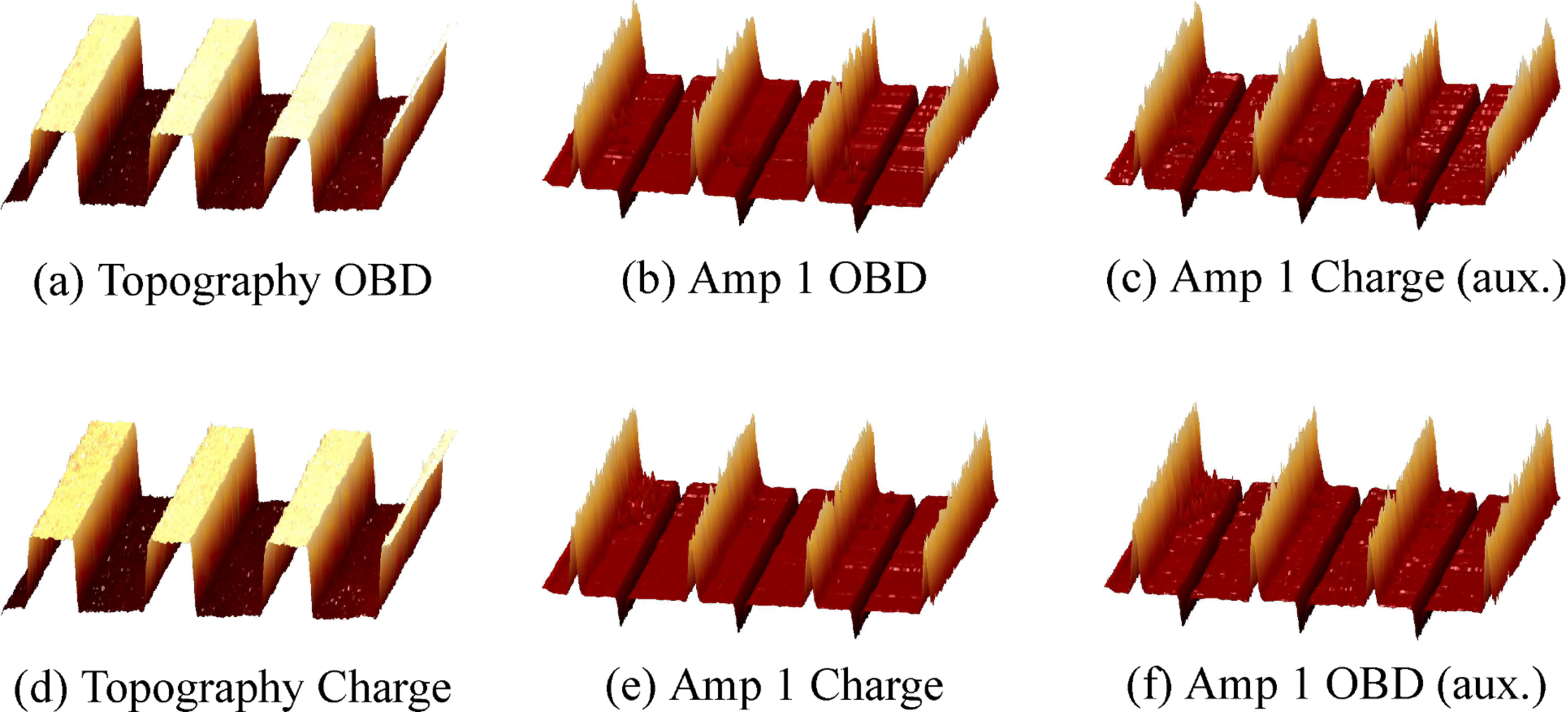
AFM Experiment on a TGZ1 calibration grating showing the 3D images of topography and fundamental mode amplitudes for (a)–(c) using the OBD sensor for feedback and (d)–(f) the charge sensor for feedback. Note, the respective auxiliary signal plotted in the third column shows no difference to the feedback signal plotted in the second column.

The TGZ1 calibration grating was imaged alternating between the OBD sensor and the charge sensor as the topography feedback signal in order to verify the suitability of imaging with charge. It can be seen from Figure 10 that due to the excellent SNR of the charge sensor the topography obtained from either of the two methods yields identical quality. Moreover, it can be noted that for stiff samples like the TGZ1, the overall conservative interactions result in no difference between the amplitude of the actual feedback signal and the auxiliary signal (compare Figure 10b with Figure 10c and Figure 10e with Figure 10f). For both experiments, the interaction was mostly attractive as can be seen from the fundamental mode phase image (not shown) and only shows repulsive interaction at the rising edges of the features.

#### Bimodal AFM on PS/LPDE

**Figure 11:**
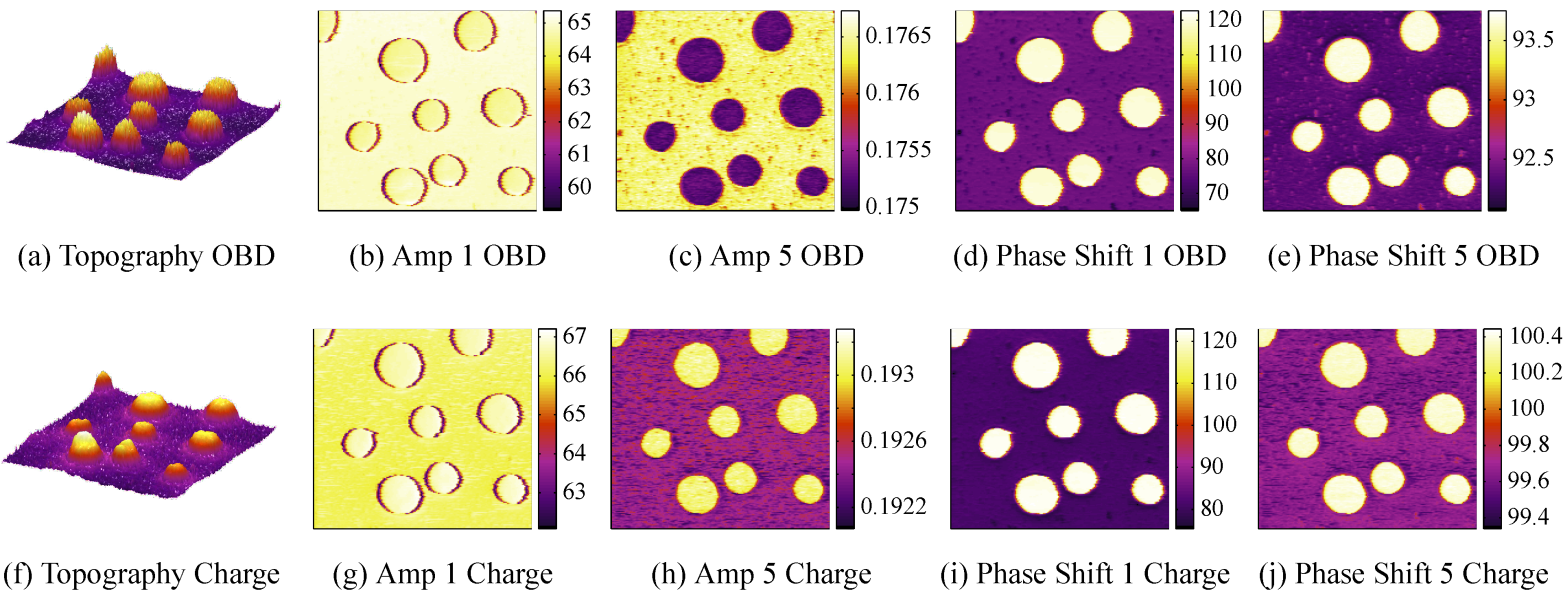
Bimodal experiment with the first and fifth eigenmode of the piezoelectric cantilever on a PS/LPDE sample showing amplitude in [nm] and phase in [°] using (a)–(e) the OBD sensor and (f)–(j) the charge sensor. Note the contrast reversal of the amplitude of the fifth mode between the OBD and charge sensor.

A two component polymer as described in section Experimental setup was imaged using bimodal AFM, i.e., by actively driving the first and fifth eigenmodes of the piezoelectric cantilever. While the *z*-axis feedback controller maintains a constant amplitude at the fundamental frequency by commanding the *z*-actuator, the higher mode is left uncontrolled and can respond freely to sample features. As such, the higher eigenmode phase contrast is often used to distinguish between material properties [37]. The experimental results are presented in Figure 11; a plane level algorithm has been applied to the topography images. The first row represents a bimodal experiment with the OBD sensor and the second row shows bimodal imaging of the same area with the charge sensor. For clarity, the phase of the first and fifth modes for each sensor have been shifted such that 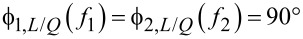 and as such 
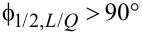
 indicates a net attractive imaging regime and 
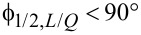
 indicates a net repulsive imaging regime. We note that the first mode interaction using either sensor is attractive on the LPDE islands and repulsive on the surrounding PS matrix. In contrast, the fifth mode interaction is consistently attractive across both features with either sensor. A clear contrast between the two polymer regions can be observed in the fifth mode phase image for either sensor. Comparing the amplitude image on the fifth mode, the contrast reversal discussed in section Approach curves is clearly visible.

## Conclusion

Experimental results using monomodal and bimodal atomic force microscopy with the first and fifth eigenmode of a piezoelectric cantilever on a variety of samples validate that the self-sensing scheme proposed in this work achieves remarkable signal-to-noise ratios and can therefore be used to provide both the feedback signal for topography imaging on the fundamental mode and phase imaging on the higher eigenmode. The charge sensor as well as the feedthrough compensation are implemented in analog using high-bandwidth surface mount components. In this approach, due to small circuit mismatches, the feedthrough has to be canceled for each mode separately to achieve the best dynamic range which is necessary for tapping-mode AFM. The inherent self-sensing capability of a single piezoelectric layer enables the omission of the commonly used optical lever method, promoting the potential downsizing of an AFM. In future work, the authors aim to extend this work to the point where quantitative material properties can be extracted using a multimode charge sensor. Furthermore, we aim to implement an automatic feedthrough compensation scheme using disturbance observer concepts which would eliminate the need for individual analog compensation circuits. Lastly, we note that not all eigenmodes are equally observable with the present cantilever geometry and location of the piezoelectric layer. In order to observe a specific higher eigenmode, a modal optimization routine should be employed which places individual piezoelectric transducers at locations where that mode shows a uniform and maximum strain distribution. Therefore, future work will aim at exploring optimal cantilever geometry and piezoelectric layer layout to maximize the deflection to strain sensitivity at each mode.
